# Case report: Gastric tube cancer after esophagectomy—Retrograde perfusion after proximal resection of right gastroepiploic artery

**DOI:** 10.1016/j.ijscr.2019.03.020

**Published:** 2019-03-26

**Authors:** Akio Sakaki, Jun Kanamori, Ataru Sato, Naoya Okada, Koshiro Ishiyama, Daisuke Kurita, Junya Oguma, Hiroyuki Daiko

**Affiliations:** Department of Esophageal Surgery, National Cancer Center Hospital, 5-1-1 Tsukiji, Chuo-ku, Tokyo, 104-0045 Japan

**Keywords:** CT, computed tomography, Gastric tube cancer, Oesophageal cancer, Right gastroepiploic artery, Gastric pull-up, Retrograde perfusion

## Abstract

**Introduction:**

We report a case of a 57-year-old patient with gastric tube cancer after subtotal esophagectomy and retrosternal gastric pull up.

**Case presentation:**

The patient developed gastric cancer 4 years after undergoing treatment for esophageal squamous cell cancer; the treatments included thoracoscopic subtotal esophagectomy, gastric pull-up reconstruction via a retrosternal route in salvage setting following definitive chemoradiation. Because the gastric tube cancer was located around the pylorus, transabdominal partial resection, which is much less invasive than total resection via sternotomy, was performed. During surgery, retrograde pulsation of the proximally resected right gastroepiploic artery was observed. Owing to an ample blood supply to the oral remnant of the gastric tube, vascular reconstruction of the right gastroepiploic artery was omitted. The postoperative recovery was eventless.

**Discussion:**

The right gastroepiploic artery is considered essential for blood supply to the gastric tube. However, there was no sign of ischemia after proximal resection of this artery, which suggests the vasculature was altered after gastric tube construction.

**Conclusion:**

This case shows that partial distal resection of the gastric tube can be performed safely without vascular reconstruction of the right gastroepiploic artery. Favorable long-term results after gastric tube reconstruction support the possibility of bilateral blood supply to the gastroepiploic arcade.

## Highlights

• Surgical treatment of post-esophagectomy gastric tube cancer is individual and challenging.
• We safely and successfully treated a gastric tube cancer via partial resection, sacrificing Right gastroepiploic artery.
• Retrograde pulsation of the right gastroepiploic artery was seen during surgery.
• Consequently, vascular reconstruction of this artery was not required.
• Bilateral vascularization may reduce the risk of perioperative complications.

## 1 Introduction

Metachronous second cancers can occur after resection of esophageal cancers. Most commonly they are squamous cell cancers of the naso- and oropharynx, followed by adenocarcinomas of the gastric tube. The estimated 10-year cumulative rate of gastric tube cancer after esophagectomy is 5.7–8.1% [1,2].

Multimodal therapy has improved the prognosis of patients with resectable esophageal cancer; however, because these patients survive longer, the incidence of the gastric tube cancer is expected to increase [3]. Following esophagectomy, annual endoscopic surveillance is important for detecting gastric tube cancer at an early stage [4,5]. Endoscopic resection with endoscopic submucosal dissection is the appropriate treatment for early-stage gastric tube tumors because of its minimal invasiveness [[6], [7], [8]], whereas surgical resection is the only option for locally advanced tumors. Oncological radicality and operative risks should be carefully weighed when making decisions about the extent of resection. If the tumor is approachable via laparotomy, partial gastric resection is preferable to sternotomy and thoracotomy, which are associated with high morbidity and mortality rates.

Perfusion of the right gastroepiploic artery is commonly considered essential for blood supply to the gastric tube. Depending on the operative findings, it may be necessary to sacrifice this artery within the distal partial resection area of the gastric tube. In such cases, reconstruction of the artery via a vascular anastomosis should be considered [9,10]. Here we present a case of gastric tube cancer, surprisingly with retrograde perfusion of the remnant right gastroepiploic artery after proximal resection.

This case has been reported in line with the SCARE criteria [11].

## 2 Presentation of case

A 57-year-old patient developed a gastric tube adenocarcinoma after oncological esophagectomy of a squamous cell cancer. The squamous cell cancer was diagnosed in the mid third of the esophagus 6 years earlier, and its initial stage was T1bN0M0. It was treated via definitive chemoradiation at 60 Gy and chemotherapy with fluorouracil and cisplatin. The chemoradiation was performed according to the protocol of the JCOG 0502 study, which is an ongoing prospective study comparing esophagectomy with definitive chemoradiotherapy for T1bN0 cancers; it includes both randomized and patient preference arms [12]. The recurrence of tumor was suspected locally 2 years after treatment and was resected by performing thoracoscopic subtotal esophagectomy and two field-lymphadenectomy in a salvage setting. Reconstruction was accomplished via gastric pull-up through the retrosternal route and cervical anastomosis.

At the annual endoscopic follow-up 4 years after esophagectomy, a tumor was detected around the pylorus of the gastric conduit. The tumor was histologically diagnosed as an adenocarcinoma, and its clinical stage based on preoperative computed tomography (CT) and endoscopic ultrasound was cT3N1M0; hence, surgical resection was indicated. A single lymphatic metastasis was suspected in the infrapyloric lymph node (lymph node station number 6a according to the Japanese classification of gastric cancer). Preoperative CT showed that the tumor was entirely within the abdomen.

**Fig. 1:**
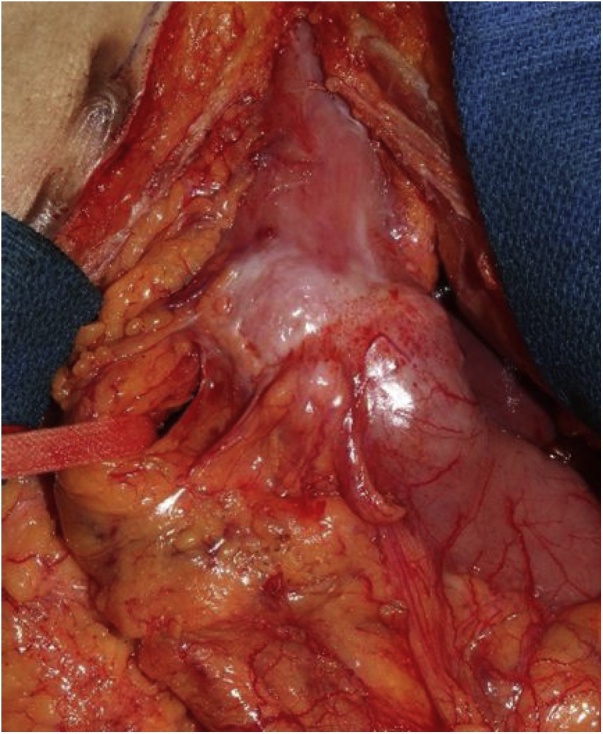
Intraoperative view of the gastric tube cancer. The right gastroepiploic artery is marked using a band.

**Fig. 2:**
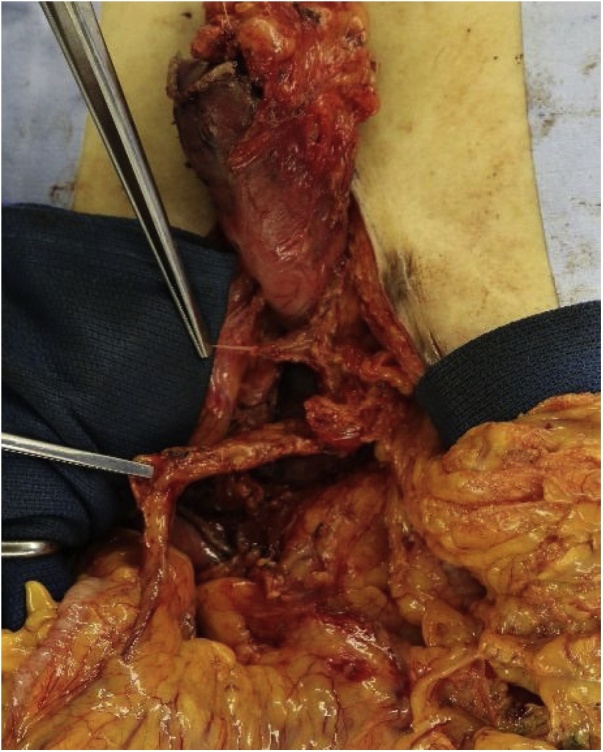
Photograph taken after distal resection of the gastric tube involving the right gastroepiploic artery. The proximal and distal resection margins of the right gastroepiploic artery are indicated by the forceps.

Resection of the proximal right gastroepiploic artery was necessary to achieve an adequate extent of lymphadenectomy, especially considering the suspected metastasis in the infrapyloric lymph node. Thus, we planned to perform distal partial resection of the gastric tube, followed by lymphadenectomy along and reconstruction of the right gastroepiploic artery. The right gastric artery was preserved during gastric tube resection (Fig. 1). After proximal resection of the right gastroepiploic artery, the remnant vessel had visible retrograde pulsation, contrary to our expectation (Fig. 2). The oral resection margin of the gastric wall had sufficient blood supply owing to retrograde perfusion. Because there was no sign of macroscopic ischemia for more than 20 min after resection of the right gastroepiploic artery, we decided to forego its reconstruction. Gastrointestinal reconstruction was performed by end-to-side gastroenterostomy in the Billroth II position.

**Fig. 3:**
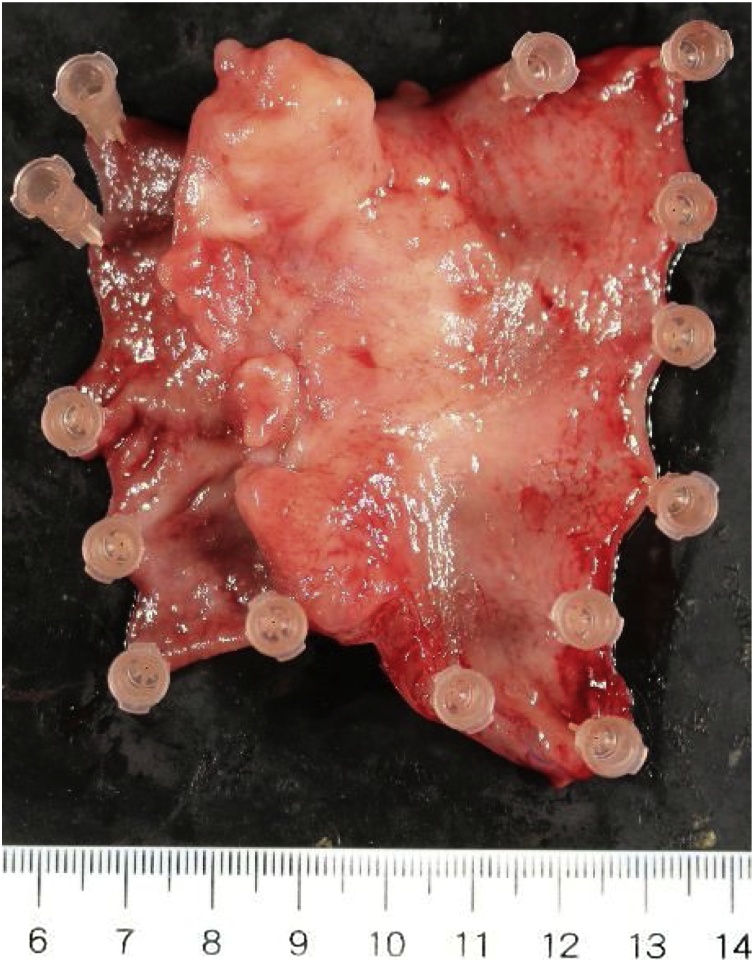
Surgical specimen of the gastric tube cancer (scale in cm.).

The postoperative course was uncomplicated. Six days after surgery, the patient began oral intake of nutrients. Eleven days after surgery, the patient was fully recovered and discharged in good clinical condition. Based on postoperative histopathological examination of a surgical specimen, the pathological stage of the gastric tube tumor was pT3N1M0 (Fig. 3). A positive extranodal lymphatic metastasis was observed in the tissue along the right gastroepiploic artery (lymph node station number 4d, which is continuous with the infrapyloric lymph node). Three months after surgery, the patient was doing well without any sign of recurrence.

## 3 Discussion

Surgical treatment of gastric tube cancer is challenging. If the tumor can be safely resected via an abdominal approach, total resection of the gastric tube should be reconsidered as it is highly invasive, with an enormous risk of life-threatening postoperative complications [13]. Sternotomy in particular has a high incidence of severe morbidities such as anastomotic leakage, sternal osteomyelitis, and colon conduit necrosis. Use of video-assisted total resection of the gastric tube as an alternative to sternotomy has been reported [14,15]. However, if technically feasible, partial resection is the best option, as it precludes severe postoperative complications and reduces perioperative risk.

Gastric tube cancer most often occurs in the distal third region of the gastric tube [16]. This location is of particular interest, because it allows the tumor to be partially resected using an abdominal approach, which is much less risky than total resection. Several diagnostic and operative strategies for preservation of the right gastroepiploic artery have been reported [17,18]. Particular to our case is definitive chemoradiation for treatment of the esophageal squamous cancer, which posed an additional risk for surgical treatment of the metachronous gastric tube cancer. Total resection of the gastric tube was a potential surgical option, as it increases the extent of lymphadenectomy for maximum oncological radicality. However, lymphadenectomy of the cranial paragastric lymph node stations (no. 1 and 2) and lesser curvature lymph node stations (no. 3a and 7) had been performed during the previous esophagectomy; hence, the benefit of radical lymphadenectomy was questionable. The extent of lymphadenectomy in cases of gastric tube cancer has not been standardized because long-term results of surgically treated gastric tube cancers are lacking.

In the present case, distal gastrectomy, including resection of the right gastroepiploic artery, did not result in insufficient perfusion of the remnant gastric tube, which is remarkable. The visible pulsation of the resected distal margin of the right gastroepiploic artery apparently reflects retrograde perfusion.

A few similar cases have been reported, in which the cranial remnant of the gastric tube survived even after proximal resection of the right gastroepiploic artery within the distal resection area of the gastric tube [19]. This phenomenon suggests that bidirectional blood supply through the gastroepiploic vessel arcade, which is similar to Riolan’s arcade, is a long-term result of collateral vascularization after subtotal esophagectomy and gastric pull-up. Two blood supply routes to the gastroepiploic arcade are conceivable: an intramural vascular network in the gastric tube and an arterial network from the omental branches. Experimental studies show that ligation of the left gastric artery after ischemic conditioning promotes neovascularization of the intramural vascular network in a time-dependent manner [20]. An intramural arterial network arising from the esophagogastric anastomosis and preserved right gastric artery might be the source of the intramural vascular network, but whether the vascular network, which is of capillary origin, emitted the retrograde pulsation of the gastroepiploic artery in our study is questionable. Another vascular structure perhaps responsible for this pulsation is the transomental arch of Haller and Barkow. This arch connects the arterial blood flow from the right omental artery to the other omental branches as collateralization of the right gastroepiploic artery [21]. Hyperplastic growth of the arch of Haller and Barkow might be facilitated by construction of a gastric tube. Collectively, the above possibilities indicate that the mechanism of long-term blood supply to the gastric tube remains to be fully clarified.

## 4 Conclusion

Our case underscores the need to reconsider our basic understanding of surgically altered vascular anatomy. To assess the safety of resection of the right gastroepiploic artery, additional information about long-term collateral vascularization after esophageal surgery is required. Our case also shows the importance of individualized surgical strategies for the treatment of gastric tube cancer. In particular, it highlights the potential of bilateral vascularization to reduce the perioperative risk of complications in patients who undergo limited resection for gastric tube cancer.

## Conflict of interest statement

The authors have no conflict of interests to disclose.

## Sources of funding

This research did not receive any specific grant from funding agencies in the public, commercial, or not-for-profit sectors.

## Ethical approval

This case report was approved by the Institutional Review Board of National Cancer Center Hospital (approved number is 2017-061) according to the ethical standards laid down in the Declaration of Helsinki.

## Consent

Written informed consent was obtained from the patient.

## Author’s contribution

A Sakaki wrote the main manuscript body.

Case report concept, design was given by A Sakaki and H Daiko.

H Daiko, J Kanamori and A Sato performed the surgery. A Sakaki, J Kanamori, N Okada, K Ishiyama, A Sato, D Kurita, J Oguma and H Daiko participated the pre- and postoperative treatment of the patient. J Oguma, J Kanamori, A Sato and H Daiko read the article critically and gave the first author suggestions to improve the manuscript. Administrative, technical or material support was also given by J Kanamori, J Oguma, N Okada, K Ishiyama, A Sato, D Kurita.

## Registration of research studies

This case report was approved by the Institutional Review Board of National Cancer Center Hospital (approved number is 2017-061) according to the ethical standards laid down in the Declaration of Helsinki.

## Guarantor

The Guarantor of this case report is H Daiko.

## Provenance and peer review

Not commissioned, externally peer-reviewed.
